# Cigarette Smoke Suppresses the Surface Expression of *c-kit* and Fc**ε**RI on Mast Cells

**DOI:** 10.1155/2013/813091

**Published:** 2013-02-12

**Authors:** M. E. Givi, B. R. Blokhuis, C. A. Da Silva, I. Adcock, J. Garssen, G. Folkerts, F. A. Redegeld, E. Mortaz

**Affiliations:** ^1^Division of Pharmacology, Utrecht Institute for Pharmaceutical Sciences, Faculty of Science, Utrecht University, 3584 CG Utrecht, The Netherlands; ^2^Integrative Pharmacology, Department of Biosciences, AstraZeneca R&D Lund Respiratory and Inflammation Research Area, 22 187 Lund, 43183 Mölndal, Sweden; ^3^Airways Disease Section, National Heart and Lung Institute, Imperial College London, South Kensington Campus, London SW7 2AZ, UK; ^4^Danone Research-Centre for Specialised Nutrition, P.O. Box 7005, 6700 CA Wageningen, The Netherlands; ^5^Department of Immunology, Chronic Respiratory Disease Research Center and National Research Institute of Tuberculosis and Lung Disease (NRITLD), Masih Daneshvari Hospital, Shahid Beheshti University of Medical Sciences, P.O. Box 19575/154, Tehran, Iran

## Abstract

Chronic obstructive pulmonary disease (COPD) is a multicomponent disease characterized by emphysema and/or chronic bronchitis. COPD is mostly associated with cigarette smoking. Cigarette smoke contains over 4,700 chemical compounds, including free radicals and LPS (a Toll-Like Receptor 4 agonist) at concentrations which may contribute to the pathogenesis of diseases like COPD. We have previously shown that short-term exposure to cigarette smoke medium (CSM) can stimulate several inflammatory cells via TLR4 and that CSM reduces the degranulation of bone-marrow-derived mast cells (BMMCs). In the current study, the effect of CSM on mast cells maturation and function was investigated. Coculturing of BMMC with CSM during the development of bone marrow progenitor cells suppressed the granularity and the surface expression of *c-kit* and Fc**ε**RI receptors. Stimulation with IgE/antigen resulted in decreased degranulation and release of Th1 and Th2 cytokines. The effects of CSM exposure could not be mimicked by the addition of LPS to the culture medium. In conclusion, this study shows that CSM may affect mast cell development and subsequent response to allergic activation in a TLR4-independent manner.

## 1. Introduction

The incidence of chronic respiratory diseases like chronic obstructive pulmonary disease (COPD) and asthma is increasing dramatically and currently affect the lives of approximately 300 and 200 million people, respectively, worldwide [1, 2]. COPD is characterized by a complex interaction between inflammatory and structural cells, all of which have the capacity to release multiple inflammatory mediators [3]. Cigarette smoke (CS) is the major player in the pathogenesis of COPD [3]. Exposure to CS activates an inflammatory cascade in the airways resulting in the production of a number of potent cytokines and chemokines with accompanying damage to the lung epithelium, increased permeability, and recruitment of macrophages and neutrophils [4]. CS contains high levels of reactive oxygen species [5] and LPS [6, 7]. LPS is a strong Toll-Like Receptor (TLR) 4 agonist [8]. TLRs are an evolutionarily conserved family of cell surface molecules which participate in innate immune response [9, 10]. The effects of smoking on inflammatory cell maturation and differentiation have not been well described. Upon encountering pathogens and/or proinflammatory mediators, cells undergo a transformation process termed “maturation,” which, for example, enhances dendritic cell (DC) Ag-presenting capacity or ability to release inflammatory cytokines. The role of TLRs in maturation and development of DCs [11] and B cells [12] has been extensively described. In this regard, it has been reported that CS exposure leads to (a) decreased sputum mature DCs in healthy smokers and patients with COPD [13], (b) impaired DC maturation and T-cell proliferation in thoracic lymph nodes of mice [14] and (c) suppressed generation of IL-12 and IL-23 from DCs mediated through ERK-dependent pathways [15]. 

Parental smoking during childhood and personal cigarette smoking in teenage and early adult life are associated with a lower risk of allergic sensitization in those with a family history of atopy. The underlying mechanisms for this association remain to be determined, but the findings are consistent with the hypothesis that the immune-suppressant effects of CS protect against atopy [16]. So far few studies have reported on the role of mast cells in human smokers and in animal models of emphysema. Mast cells normally reside close to epithelia, blood vessels, nerves, smooth muscle cells, and mucus-producing glands [17]. Mast cells play a crucial role in allergic reactions [18]. Interestingly, emerging evidence also suggests a role of mast cells in the pathogenesis of emphysema [19, 20]. Kalenderian et al. [19, 20] found that the levels of mast cell mediators, such as histamine and tryptase, are considerably elevated in BALF from smokers. The importance of mast cells is further supported by the fact that mast cell tryptase activity is correlated with the severity of COPD [21], and in COPD patients an accumulation of mast cells in the airways has been observed [22]. Mast cells located here could be exposed to inhaled environmental challenges, and mast cell activation results in the coordinated release of proinflammatory mediators into the surrounding tissue; activation of this cell type may result in pathology associated with chronic inflammatory stimuli [23, 24]. Mast cells play a crucial role in acute and allergic inflammation and have Fc*ε*RI on their surface [24]. Cross-linking of surface IgE molecules results in exocytosis of preformed mediators such as amines and proteases, as well as the release of newly generated mediators including leukotrienes, prostaglandins, and a variety of cytokines such as Th1 (TNF-*α*, IL-6) or Th2 cytokines (IL-13, IL-4, IL-5) [24]. 

Previously, we showed that CSM (without IgE/Ag activation) does not trigger degranulation of bone marrow-derived mast cells (BMMCs) but does induce the release of chemokines [2]. In addition, CSM exposure suppresses IgE-mediated mast cell degranulation and cytokine release but had no effect on leukotriene release. This suggests that exposure to CSM may lead to a reduced allergic activation of mast cells without affecting their response to other stimuli [25]. In contrast, CS exposure in vivo enhanced OVA-specific IgE levels, Penh values, and recruitment of inflammatory cells including mast cells in OVA-exposed allergic mice [26]. 

In the current study, we investigated the effect of CSM exposure on the mast cell development from bone marrow progenitor cells.

## 2. Materials and Methods

### 2.1. Reagents

Recombinant mouse IL-3 and SCF (stem cell factor) were purchased from PeproTech (tebu-bio, Heerhugowaard, The Netherlands). LPS (*Escherichia coli *055.B5) was purchased from Sigma (Sigma-Aldrich, Zwijndrecht, The Netherlands). RPMI 1640, Tyrode's buffer, fetal calf serum, nonessential amino acids were purchased from GIBCO BRL Life Technologies (GIBCO-BRL Invitrogen Corporation, Carlsbad, CA, USA). Penicillin, streptomycin, L-glutamine, sodium pyruvate, and 2-mercaptoethanol were obtained from Sigma-Aldrich. 

### 2.2. Production of Cigarette Smoke Medium (CSM)

Cigarette smoke-conditioned medium (CSM) was produced as described previously [25]. CSM was generated by burning reference cigarettes 2R4F (University of Kentucky, Lexington, KY), using the TE-10z smoking machine (Teague Enterprises, Davis, CA, USA), which is programmed to smoke cigarettes according to the Federal Trade Commission protocol (35 mL puff volume drawn for 2 s, once per minute). Briefly, this machine was used to direct main- and side-stream smoke from one cigarette through a 5 mL culture medium (RPMI without phenol red). Hereafter, absorbance was measured spectrophotometrically, and the media were standardized to the absorbance at 320 nm. The pH of the resultant extract was titrated to pH 7.4 and diluted with medium. This concentration (optical density [OD] = 4.0) was serially diluted with untreated media to 0.75%, 1.5%, and 3% OD and used in the indicated experiments. In preliminary experiments, a CSM concentration of 1.5% was found optimal in the culture experiments.

### 2.3. Mouse Bone Marrow-Derived Mast Cell (BMMC) Cultures and CSM Treatment

BMMCs were generated from bone marrow of male BALB/cBy mice as described previously [27]. Cells were cultured in RPMI medium supplemented with mitogen-stimulated spleen cell conditioned medium (see below) [28]. 

 Cells were used for the experiments after 3 weeks when a mast cell purity >95% was achieved. Bone marrow cells were cocultured (1 × 10^6^/mL) with either 1.5% CSM or LPS (100 ng/mL) during the third week of culture. 

### 2.4. Pokeweed Mitogen-Stimulated Spleen Cell Conditioned Medium (PWM-SCM)

Spleen cells from BALB/c mice (Charles River Breeding Laboratories) were cultured at a density of 2 × 10^6^ cells/mL in RPMI 1640 medium containing 4 mM l-glutamine, 5 × 10^−5^ M 2-mercaptoethanol, 1 mM sodium pyruvate, 100 U/mL penicillin, 100 *μ*g/mL streptomycin, and 0.1 mM nonessential amino acids (complete RPMI 1640) containing lectin (8 *μ*g/mL) and placed in 75 cm^2^ tissue culture flasks. The cells were incubated at 37°C in a 5% CO_2_ humidified atmosphere. After 5–7 days, medium was collected, centrifuged for 15 min at 3200 ×g, filtered through a 0.22 *μ*m Millipore filter, and used as PWM-SCM.

### 2.5. Toluidine Blue Staining

The granularity of the mast cells was determined by toluidine blue staining [29]. In brief, the cells were cytospun, fixed with Carnoy's fluid, and then stained by either 2 minutes with acid toluidine blue (pH = 2.7). Cells were examined by light microscopy. 

### 2.6. Mast Cell Degranulation Assay

*The degranulation assay was performed as described before [28]*. Briefly, approximately 2-3 × 10^6^ cells from each group were resuspended in culture medium (enriched medium) and incubated with 1 *μ*g/mL anti-DNP-IgE for 2 hr. After that, cells were washed and resuspended at a density of 0.6 × 10^6^ cells/mL. Cells were aliquoted in 96 well plates (3 × 10^4^ cells per well) and activated with indicated concentrations of DNP-conjugated ovalbumin (DNP-Ova) for 30 min. After incubation, supernatants were collected. Cells were subsequently lyzed using 0.1% NP-40 (Pierce) in order to quantify the total *β*-hexosaminidase activity present in these cells. Samples were incubated with 4-methylumbelliferyl glucosaminide (4-MUG) (Sigma) in 0.1 M citrate buffer (pH 4.5) for 1 h at 37°C. 4-MUG hydrolysis was determined by fluorimetric measurement (*λ*ex: 360 nm, *λ*em: 452 nm) using a Millipore Cytofluor 2350 microplate reader. The percentage of *β*-hexosaminidase released was calculated by determining the ratio of fluorescence supernatant/fluorescence cell lysate corrected for the *β*-hexosaminidase activity present in the supernatant of nonchallenged cells.

### 2.7. Flow Cytometry Analysis

BMMCs were harvested, and after washing with cold PBS, the cell-surface Fc receptors were blocked with 2.4G2 (PharMingen, San Diego, CA, USA) before staining. We used a PE-conjugated anti-mouse *c-kit* (PharMingen) to stain *c-kit,* and mouse Fc*ε*RI was stained with an FITC-conjugated anti-mouse Fc*ε*RI antibody (PharMingen) and compared with matched isotype control antibodies. The cells were incubated with antibodies in 50 *μ*L of PBS for 1 h at 4°C, washed with PBS, and analyzed on an FACSCantoII flow cytometer (Becton Dickinson, San Jose, CA, USA). Dead cells were gated out when performing the analysis.

### 2.8. Measurement of Cytokines

*Briefly*, approximately 1 × 10^6^ cells for each experimental condition were resuspended in culture medium and incubated with 1 *μ*g/mL anti-DNP IgE for 2 hr. After that, cells were washed and resuspended. Cells were aliquoted in 96 well plates (1 × 10^6^ cells/mL) and activated with indicated concentrations of DNP-Ova for 16 h. IL-4, IL-5, IL-6, IL-13, and TNF-*α* concentrations in cell supernatants were quantitated using ELISA (Invitrogen and eBioscience), according to the manufacturer's instructions. 

### 2.9. Statistical Analysis

Experimental results are expressed as mean ± S.E.M. Results were tested statistically by an unpaired two-tailed Student's *t*-test or one-way ANOVA, followed by a Newman-Keuls test for comparing all pairs of groups. Analyses were performed by using GraphPad Prism (version 5.04). Results were considered statistically significant when *P* < 0.05. 

## 3. Results

### 3.1. CSM Reduced the Granularity of Mast Cells during Culturing

**Figure 1:**
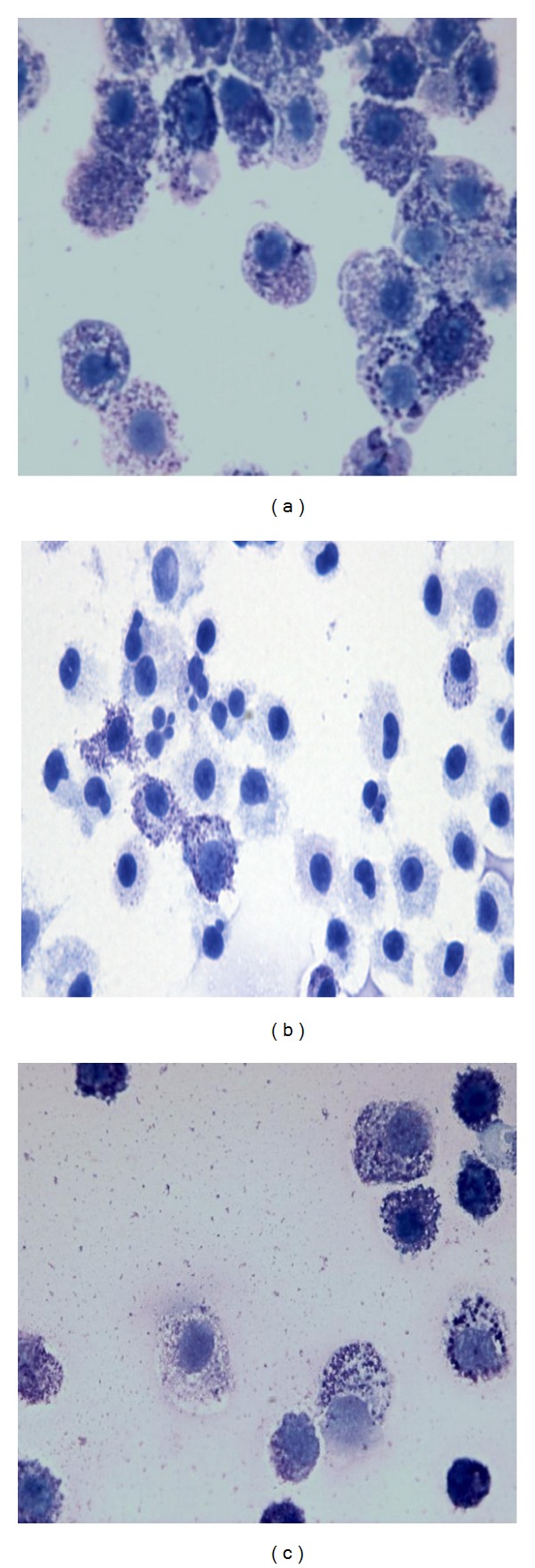
Long-term culture in presence of CSM reduces the density of mast cell granules. BMMCs from BALB/c mice were cultured in presence with medium only (a), CSM (1.5%) (b), or LPS (c) (1 *μ*g/mL) during the third week of culturing bone marrow cells as described in Section 2. Cells were stained with toluidine blue.

Bone marrow cells were cultured with CSM or LPS during the third week of mast cell development. Cell granularity was analyzed by staining with toluidine blue (Figure 1(a)). Coculturing cells with CSM (1.5%) decreased the granularity of mast cells (Figure 1(b)). LPS (1 *μ*g/mL) did not affect the granularity of cultured mast cells (Figure 1(c)). 

### 3.2. CSM Decreased *c-Kit* (CD117) and Fc*ε*RI Expression on Mast Cells

**Figure 2:**
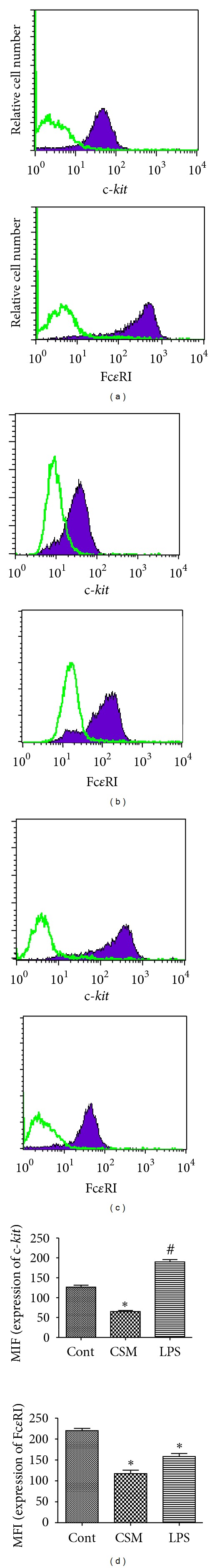
CSM modulates surface expression of Fc*ε*RI and *c-Kit*. BMMCs were cocultured in presence or absence of CSM (1.5%) or LPS during the third week of bone marrow culture. The surface expression of *c-kit* (upper panels) and Fc*ε*RI (lower panels) was detected by flow cytometry (blue histograms): control (a), CSM (b), and LPS (c). Green histograms represent isotype controls. (d) Quantification of 3 representative FACS analyses showing the mean fluorescence intensity (MFI) for each group. Values are expressed as mean ± S.E.M (*n* = 3). ^∗, #^ *P* < 0.05 is significantly different (increased/decreased) compared to control.

Mast cell *c-kit* and Fc*ε*RI surface expression was determined after CSM exposure using flow cytometry. Coculture with CSM significantly suppressed the surface expression of *c-kit* and Fc*ε*RI on mast cells (Figure 2: upper and lower panels, control (a), and CSM (b)). In contrast, longterm culture with LPS did not change the surface expression of *c-kit* and Fc*ε*RI (Figure 2(c) upper and lower panels). FACS data of 3 representative experiments were quantified in (d) showing the mean fluorescence intensity (MFI) for each experimental group. Cell viability was not affected by either CSM or LPS treatment (data not shown). 

### 3.3. Long-Term Exposure to CSM Modulates IgE/Ag-Mediated Degranulation and Cytokine Production by Mast Cells

**Figure 3:**
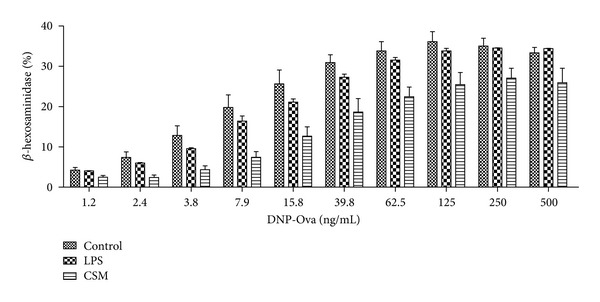
Long-term exposure of mast cells to CSM inhibits allergic degranulation. BMMCs were cultured in regular culture medium, in the presence of CSM (1.5%) or LPS (100 ng/mL) in the third week of bone marrow culture. Then, cells were sensitized with DNP-specific IgE, followed by activation with dinitrophenyl-conjugated human ovalbumin (DNP-Ova). Degranulation was assessed by the release of *β*-hexosaminidase in the supernatants from cells. Data are mean ± SEM of quadruplicate samples (*n* = 4).

Stimulation with IgE/Ag caused a dose-dependent degranulation of mast cells in the control group (Figure 3). Coculturing mast cells with CSM (1.5%) reduced IgE-mediated degranulation (Figure 3). In contrast, LPS, a TLR4 agonist, did not affect IgE/antigen-induced BMMC degranulation (Figure 3). 

**Figure 4:**
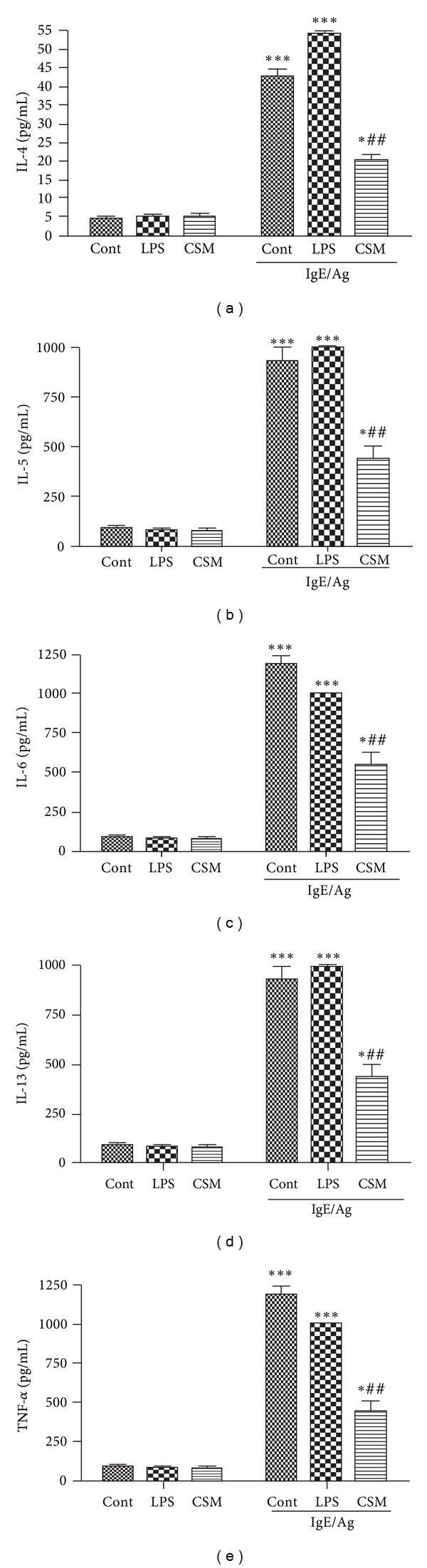
Long-term exposure of mast cells to CSM inhibits cytokine production by mast cells. BMMCs (control, CSM, and LPS cultured for 4–6 days) were sensitized with DNP-specific IgE, followed by activation with dinitrophenyl-conjugated ovalbumin (DNP-Ova) for 9 h. The levels of cytokines IL-4 (a), IL-5 (b), IL-6 (c), IL-13 (d), and TNF-*α* (e) in the supernatants were estimated by ELISA. Data are mean ± SEM of quadruplicate samples. The asterisks represent significant differences between nonactivated and activated cells (***P < 0.001). Hatches represent significant differences between control mast cells and mast cells cocultured with CSM or LPS (^#^ *P* < 0.05 and ^##^ *P* < 0.001).

CSM significantly suppressed the IgE-receptor mediated production of IL-4, IL-5, IL-6, IL-13, and TNF-*α* by mast cells (Figures 4(a)–4(e)). Culturing with LPS did not significantly change IL-4, IL-5, IL-6, IL-13, and TNF-*α* production (Figures 4(a)–4(e)). 

## 4. Discussion

In the current studies, we further investigated the effects of CSM on the development and function of primary cultured bone-marrow-derived mast cells. We show that BMMC exposed to CSM during development from progenitor cells inhibited mast cell development as determined by toluidine staining and expression on *c-kit* and Fc*ε*RI. Furthermore, the release of both Th1 and Th2 cytokines in response to Fc*ε*RI activation was reduced. Interestingly, the TLR4 agonist LPS did not affect these parameters and even slightly increased IL-4 production. 

Mast cells are important in allergic airway diseases, but they have remained poorly studied in nonallergic inflammatory airway diseases like COPD. Mast cells are of particular interest due to their ability to promote airway remodeling and mucus hypersecretion. Clinical data show increased levels of mast cell tryptase and degranulated mast cells in the lavage and bronchial tissue of smokers [30–33]. Moreover, CS exposure facilitates allergic sensitization in mice [34].

We have previously reported that short-term exposure of mature mast cells to CSM attenuated their response to allergic stimuli [28]. Kim et al. also showed an inhibitory effect of CSM on mast cell activation which suggests that CS may contribute to the reduced allergic symptoms observed in smokers [26].

Mast cells are functionally dynamic effector cells in innate and adaptive immunity [24]. Two mast cell surface receptors *c-Kit* and Fc*ε*RI mediate activation via innate and adaptive immune mechanisms, respectively [17, 23, 24]. *c-Kit* is expressed on both mature mast cells and on the earliest mast cell progenitors [35, 36]. *c-Kit* is expressed both as a soluble form and on the cell membranes [36]. Although *c-Kit* represents a major growth and differentiation factor for both murine and human mast cells [35, 37] it also promotes *c-Kit*-dependent mast cell mediator release [38] as well as the release of mast cells mediators via IgE-dependent mechanisms [39]. IgE-dependent allergic diseases are initiated by multivalent binding of allergens to IgE that is bound to Fc*ε*RI on mast cells [40]. Fc*ε*RI plays a critical role in allergic reactions. It is the major surface receptor through which mast cells direct immunologically specific secretory effects such as the release of preformed cytoplasmic granule-associated mediators and the generation and release of lipid mediators and cytokines [41]. Thus, the suppression of *c-Kit* and Fc*ε*RI on the mast cells by CSM could account for decreased responsiveness of mast cells to IgE/Ag activation. Our study suggests that LPS may not be involved in the mechanism by which CSM affects mast cell maturation and activation.

Mast cells express functional TLRs [42] which may account for the protection conferred by mast cells against bacterial and parasitic infections [43]. Activated mast cells release an array of potent inflammatory mediators by rapid discharge of preformed mediators in granules, the generation of inflammatory lipids from arachidonic acid, and the production of numerous Th2-type cytokines and chemokines [44]. All these responses are evoked by allergens via Fc*ε*RI, while stimulation of mast cells via TLR2 and TLR4 receptors results primarily in generation of cytokines such as IL-4, IL-5, IL-6, IL-10, IL-13, and TNF-*α* [43]. Saluja et al. recently showed that prolonged exposure of mast cells to LPS amplifies Fc*ε*RI-mediated degranulation, lipid mediator generation, and cytokine production [45]. This is in contrast to the effects found in this study, where LPS treatment did not increase IgE-mediated mast cell activation. The discrepancy may be due to the different mouse strains used in both studies and the time of treatment during mast cell development. 

In conclusion, our study suggests that CSM, independent of TLR4 signaling, suppresses the maturation and function of mast cells. This suppressive effect of cigarette smoke on mast cells may account for the reduced allergic response seen in animal models of cigarette-smoke-induced emphysema [46].

## Abbreviations

BMMC: Bone-marrow-derived mast cell
CSM: Cigarette smoke medium
COPD: Chronic obstructive pulmonary disease
FACS: Fluorescence-activated cells sorting
SCF: Stem cell factor
TLR: Toll-like receptor.
